# Efficiently generate functional hepatic cells from human pluripotent stem cells by complete small-molecule strategy

**DOI:** 10.1186/s13287-022-02831-1

**Published:** 2022-04-11

**Authors:** Tingcai Pan, Ning Wang, Jiaye Zhang, Fan Yang, Yan Chen, Yuanqi Zhuang, Yingying Xu, Ji Fang, Kai You, Xianhua Lin, Yang Li, Shao Li, Kangyan Liang, Yin-xiong Li, Yi Gao

**Affiliations:** 1 General Surgery Center, Department of Hepatobiliary Surgery II, Guangdong Provincial Research Center for Artificial Organ and Tissue Engineering, Guangzhou Clinical Research and Transformation Center for Artificial Liver, Institute of Regenerative Medicine, Zhujiang Hospital, Southern Medical University, Guangzhou , Guangdong China; 2 Key Laboratory of Regenerative Biology, South China Institute for Stem Cell Biology and Regenerative Medicine, Guangdong Provincial Key Laboratory of Biocomputing, Guangzhou Institutes of Biomedicine and Health, Chinese Academy of Sciences, Guangzhou, 510530 China; 3 Guangdong Key Laboratory of Non-Human Primate Models, Guangdong-Hongkong-Macau Institute of CNS Regeneration, Jinan University, Guangzhou, Guangdong China; 4 State Key Laboratory of Organ Failure Research, Southern Medical University, Guangzhou, China

**Keywords:** Hepatic differentiation, Small molecule, Human pluripotent stem cells, Hepatoblasts, Hepatocyte-like cells

## Abstract

**Background:**

Various methods have been developed to generate hepatic cells from human pluripotent stem cells (hPSCs) that rely on the combined use of multiple expensive growth factors, limiting industrial-scale production and widespread applications. Small molecules offer an attractive alternative to growth factors for producing hepatic cells since they are more economical and relatively stable.

**Methods:**

We dissect small-molecule combinations and identify the ideal cocktails to achieve an optimally efficient and cost-effective strategy for hepatic cells differentiation, expansion, and maturation.

**Results:**

We demonstrated that small-molecule cocktail CIP (including CHIR99021, IDE1, and PD0332991) efficiently induced definitive endoderm (DE) formation via increased endogenous TGF-β/Nodal signaling. Furthermore, we identified that combining Vitamin C, Dihexa, and Forskolin (VDF) could substitute growth factors to induce hepatic specification. The obtained hepatoblasts (HBs) could subsequently expand and mature into functional hepatocyte-like cells (HLCs) by the established chemical formulas. Thus, we established a stepwise strategy with complete small molecules for efficiently producing scalable HBs and functionally matured HLCs. The small-molecule-derived HLCs displayed typical functional characteristics as mature hepatocytes in vitro and repopulating injured liver in vivo.

**Conclusion:**

Our current small-molecule-based hepatic generation protocol presents an efficient and cost-effective platform for the large-scale production of functional human hepatic cells for cell-based therapy and drug discovery using.

**Supplementary Information:**

The online version contains supplementary material available at 10.1186/s13287-022-02831-1.

## Background

Developing efficient and cost-effective technology to generate large quantities and high-quality functional human hepatic cells is essential for applying hepatic cells to cell-based therapy and drug toxicity screening. To date, protocols have been developed to generate hepatic cells from hPSCs, while most of the reported protocols relied on expensive growth factors, such as Activin A, BMPs, FGFs, and HGF [1–5]. This leads to critical challenges such as high cost and low reproducibility. In contrast, small molecules offer an attractive alternative to growth factors since they are usually more economical, relatively more stable, and possibly more efficient for generating hepatic cells from hPSCs [6–8]. Thus, substituting growth factors with small molecules and optimizing the hepatic generation method could contribute to scalable and integrated producing functional hepatic cells, improving cost-effectiveness and reproducibility.

Previous efforts have been made to identify small molecules that support the differentiation and maintenance of hepatic cells, and impressive advances have been undertaken to identify numerous chemical candidates [9–11]. Our previous study established a synergistic cocktail to regulate BMP, Wnt, Hedgehog, and other signaling pathways, creating a delicate balance for HBs expansion and bipotency maintenance [12]. Based on this, we further created a robust and cost-effective culture condition for the generation of self-renewing HBs and functional mature HLCs, using a small-molecule cocktail in a defined culture system [13]. Further use of a small-molecule protocol to direct hPSCs differentiation into HBs and establish a complete small-molecule formula to generate expanded HBs and functional mature HLCs efficiently would benefit many hepatic cell potential clinical applications, including cell transplantation, bio-artificial liver, drug development, and disease modeling.

So far, complete small-molecule-derived HLCs protocols have been reported in several works of the literature, with a lower hepatic differentiated efficiency than growth-factor-based approaches [6, 14–16]. This may be due to the indistinct contribution of exogenous and endogenous signals involved in hepatic differentiation. More importantly, these studies are generally characterized by hepatic marker genes and proteins expression and albumin secretion and glycogen uptake while lacking hepatocyte-specific functional analyses in vitro and in vivo. Thus, a more detailed understanding of the precise regulatory mechanisms of hepatic differentiation is vital to establishing a small-molecule-based induction protocol. Also, further efforts are needed to optimize the current chemical differentiation procedure for efficient and robust differentiation hPSCs into functional hepatic cells.

In the present study, we focused on improving chemical strategy to generate functional hepatic cells from hPSCs. We demonstrated that stepwise treatment with small molecules, including CHIR99021 (CHIR), IDE1, and PD0332991 (PD), could elevate endogenous TGF-β/Nodal signaling activity and direct hPSCs toward definitive endoderm (DE) efficiently. We further screened small molecules to identify an optimal chemical condition VDF for HBs specification. Moreover, the small-molecule-derived HBs could subsequently expand and mature into HLCs via our previously established chemical cocktail. The small-molecule-derived HLCs showed typical morphologic and functional features of mature hepatocytes, with hepatocyte-specific markers expression and functionalities in vitro and in vivo. In summary, our current chemical-based hepatic specification, expansion, and maturation method presents an efficient and robust platform for the cost-effective and large-scale production of functional human hepatic cells for clinical regenerative medicine and drug discovery applications.

## Material and methods

### Human PSCs culture and hepatic differentiation

Human PSC lines, including iPSC line (UC15) and ESC line (H1), were cultured in mTeSR1 medium (Stem Cell Technologies) on 100-fold-diluted Matrigel matrix (Growth Factor Reduced, BD Bioscience). The hPSCs colonies were passaged using Accutase (life).

**Fig. 1:**
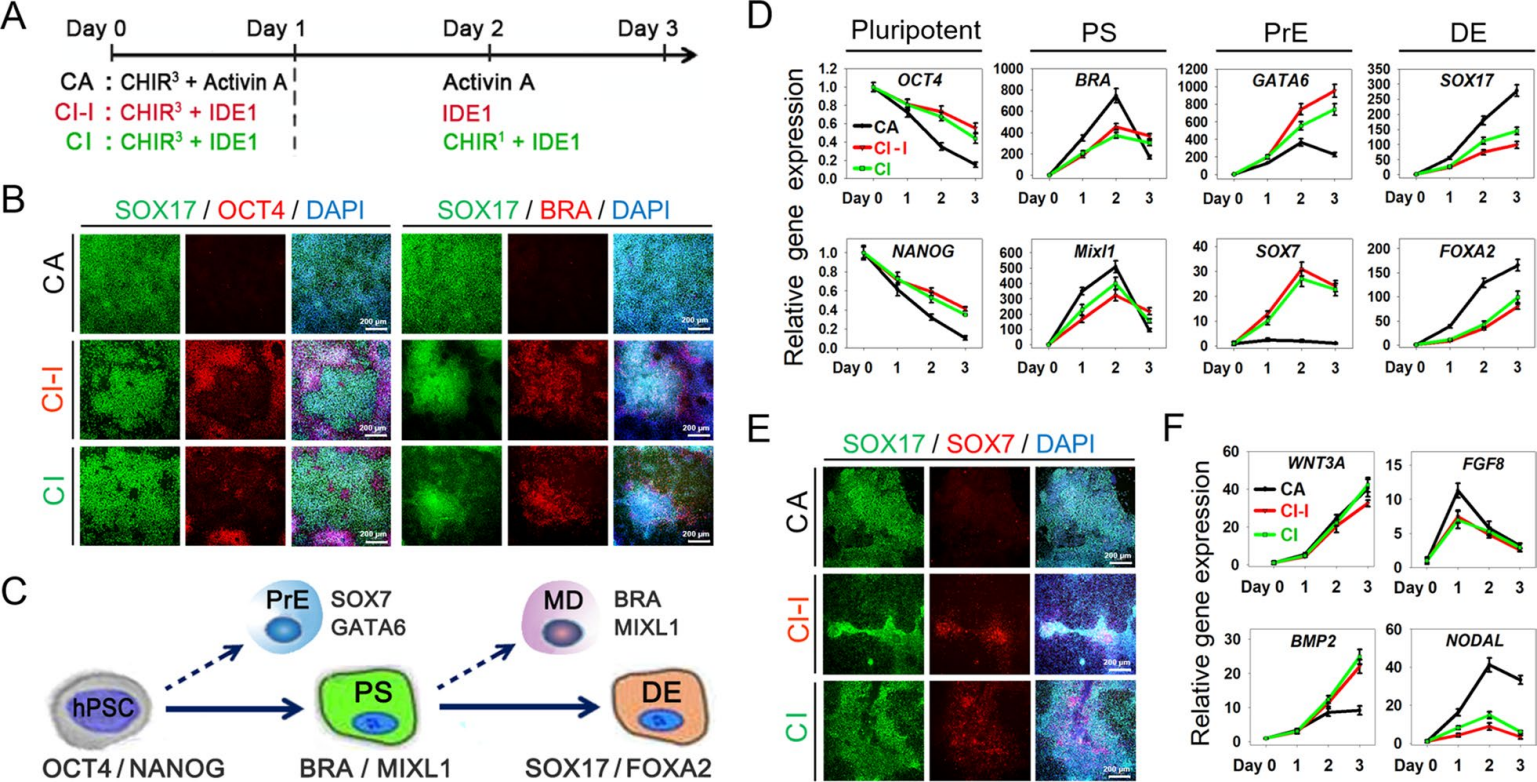
CHIR and IDE1 inefficiently induced DE differentiation from hPSCs. **A** Schematic of the two-step chemical strategy to differentiate hPSCs into DE cells. CHIR^3^: 3 µM CHIR99021, CHIR^1^: 1 µM CHIR99021. **B** Immunostaining analyzed SOX17, OCT4, and BRA expression in the differentiated cells. Scale bars 200 μm. **C** Overview of lineage relationships during DE differentiation from hPSCs. PS: primitive streak, PrE: primitive endoderm, MD: mesoderm, DE: definitive endoderm. **D** Quantitative RT-PCR analysis lineage relative genes expression of differentiated cells during DE induction. Data are presented as mean ± SEM, n = 3. **E** Immunostaining analyzed SOX17 and SOX7 expression in the differentiated cells. Scale bars 200 μm. **F** Quantitative RT-PCR analyzed signaling pathway-related genes expression patterns in DE induction with different protocols. Data are presented as mean ± SEM, n = 3

**Fig. 2:**
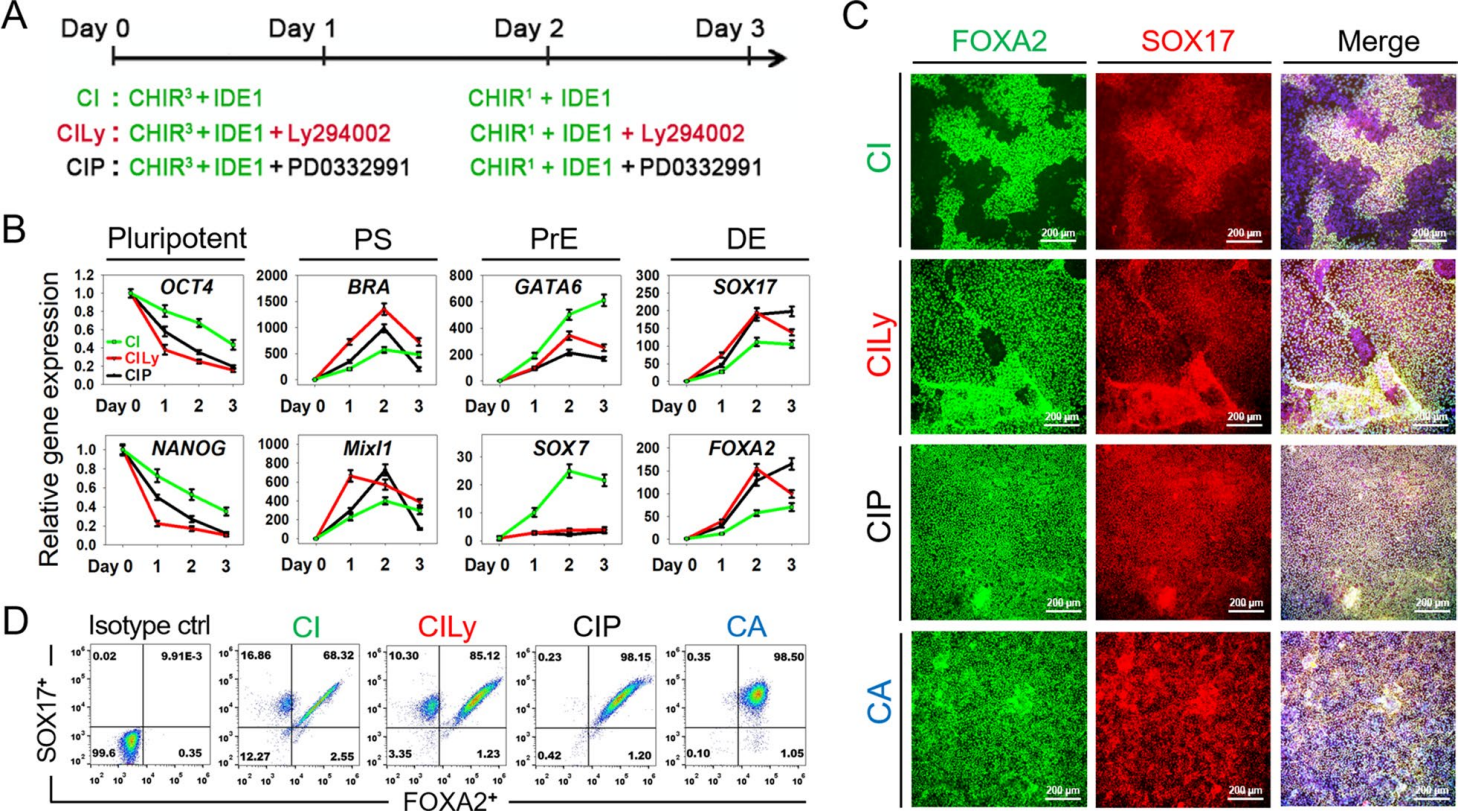
Manipulating small-molecule cocktails to improve DE differentiation. **A** Schematic of the strategy to differentiate hPSCs into DE cells with small molecules. **B** Quantitative RT-PCR analysis lineage relative genes expression of differentiated cells during DE induction with different protocols. Data are presented as mean ± SEM, n = 3. **C** Immunostaining analyses of SOX17 and FOXA2 expression in DE cells derived from different protocols. Scale bars 200 μm. **D** The efficiency of SOX17 and FOXA2 expression after different cocktails treatment, determined by the FACS examination in differentiated cells

For hepatic differentiation, we started DE induction first. Briefly, while hPSCs reached nearly 70% confluence, the medium was replaced with RPMI1640 (Gibco) supply with 1 × B27 (minus insulin, Invitrogen), containing CHIR, IDE1, 2 µM Ly294002 (Ly), and 0.75 µM PD as indicated in Figs. 1A and 2A for 3 days. Further DE specifying to HBs, DE cells were cultured in differentiation medium (RPMI1640 supply with 1 × B27 supplement [Invitrogen]), containing growth factors (20 ng/mL BMP2 and 30 ng/mL FGF4) or small molecules (10 μg/mL Vitamin C, 0.1 µM Dihexa, and 10 μM Forskolin) as indicated in Fig. 4A.

### Human HBs culture and hepatic maturation

HBs were maintained in expansion medium as described previously [12]. Briefly, the purified HBs were cultured in expansion basal medium (RPMI1640, 1 × B27 supplement, 1 × ITS) supplemented with chemical cocktails (ACDFSV), including 5 µM A8301, 3 µM CHIR, 0.1 µM Dihexa, 10 μM Forskolin, 0.5 µM SAG, and 10 μg/mL Vc. Used chemical compounds were purchased from Selleck except indicated. For cell replating in our current method, the expanding HBs were dissociated by Accutase (life) and replated on Matrigel Matrix pre-coated plates.

For hepatocyte maturation, HBs were cultured in hepatoZYME-SFM (Gibco) medium supply with 1X GlutaMAX, containing small molecules, including 5 µM A8301, 0.1 µM dexamethasone (Dex), 0.1 µM Dihexa, and 0.5 mM NH4Cl for 5 days. The medium was changed daily during the differentiation period. Used small molecules were purchased from Selleck, and growth factors were purchased from PeproTech except that indicate.

### *Functional analyses of differentiated hepatocyte *in vitro

For the urea secretion assay, the culture media of 24 h incubated in differentiated cells were collected and stored at − 80 °C. Urea concentration was analyzed by LC/MS/MS API3000 and normalized with total cell protein concentration.

To evaluate the CYP450 activity, cultures were incubated with conventional probe substrates (CYP3A4: 6 μM midazolam, CYP2C9: 10 μM diclofenan, CYP2D6: 10 μM dextromethorphan), respectively, for quantifying metabolite production. After 2 h of exposure, the culture medium was collected and stored at − 80 °C subsequently, and CYP450 activity was analyzed by LC/MS/MS API3000. Metabolite products were normalized to total cell protein.

To evaluate the glycogen production and storage ability, periodic acid-Schiff (PAS) staining was performed. According to the manufacturer’s instructions, the cultured cells were fixed with 4% PFA for 30 min, and intracellular glycogen was stained using a PAS staining solution (Muto Pure Chemicals).

For cellular indocyanine green (ICG) uptake assay, differentiated cells were incubated in media supplemented with ICG (final concentration of 1 mg/ml, Sigma-Aldrich) for 1 h at 37 °C. Cells were then washed with PBS and imaged using a phase-contrast microscope (X71, Olympus).

For cellular LDL uptake assay, the HLCs were cultured with a medium containing Alexa-Flour 488-ac-LDL for 1 h, and immunohistochemistry was performed. Nuclei were counterstained with DAPI.

### Animal model and hepatic cells transplantation

Immune-deficient NOD-SCID-IL2RG^−/−^ mice (NSI mice, GIBH) were used as recipients of human hepatic cells. Before hepatic cells transplantation, 8-week-old NSI mice received DMN intraperitoneal injections (7 mg/kg, Sigma, 1.0% dissolved in saline) for 2 consecutive days for inducing acute liver injury. Two days later, 1 × 10^6^ hepatic cells were intrasplenically transplanted into the DMN-treated NSI mice. To monitor the transplantation state, recipient mice livers were harvested at different time points after hepatic cells transplantation. All animal experiments were approved by the Animal Welfare Committee of GIBH. All protocols were approved by the appropriate Institutional Animal Care and Use Committee (IACUC).

### Quantitative RT-PCR analysis

According to the manufacturer’s protocol, total RNA was extracted using TRIzol reagent (Invitrogen) and quantified with NanoDrop 2000 (Thermo Fisher). cDNA was reverse transcribed from 2 μg RNA using ReverTra Ace (Toyobo) and oligo-dT (Takara). Quantitative RT-PCR was performed with the CFX96 machine (Bio-Rad) and SYBR Green Premix (Bio-Rad) following the manufactures’ manual. The GAPDH was used for quantitative RT-PCR normalization, and the experiments have repeated a minimum of three times to confirm the results. Primer sequences are listed in the Additional file 2: Table S1.

### Immunohistochemistry staining

Cells were washed with PBS and fixed with 4% PFA (Sigma-Aldrich) for 30 min at room temperature. After washing the cells with PBS, cells were permeabilized with 0.1% Triton X-100 in PBS for 30 min and blocked with PBS containing 5% normal goat or donkey serum for 30 min at room temperature. The primary antibodies were diluted with blocking solution and incubated at 4 °C for overnight. After washing the cells with PBS, cells were then stained with compatible Alexa Fluor-conjugated secondary antibodies in blocking solution for 1 h at room temperature. Nucleus stained with 5 μg/mL DAPI (Invitrogen). Imaging was performed on a Zeiss LSM 710 confocal microscope. The primary antibodies and secondary antibodies are described in the Additional file 2: Table S2.

### Fluorescence-activated cell sorting (FACS) and flow cytometry analysis

Cells were dissociated with Accutase and then resuspended in PBS containing 3% BSA. The collected cell suspensions were stained with FITC-conjugated human Ep-CAM antibody (Milteny) and Alexa Fluor APC-conjugated antibody against human C-Kit (B56; BD Biosciences) for 30 min on ice. EpCAM^+^/C-Kit^−^ cells were sorted using a MoFloTM fluorescence-activated cell sorter. Sorted cells were seeded on Matrigel pre-coated plates and maintained in expansion basal medium containing small-molecule cocktails.

To analyze the cell proliferation, the expanded HBs were dissociated and then resuspended in PBS containing 10% FBS. The collected cell suspensions were fixed with 4% PFA (Sigma-Aldrich) and incubated in blocking and permeabilizing buffer, containing 0.1% Triton X-100, and 5% normal donkey serum in PBS for 30 min at room temperature. The cells were then incubated with APC-conjugated Ki67 (B56; BD Biosciences) and other indicated antibody for 30 min on ice. Corresponding isotype antibodies were used as controls. Flow cytometry analyses were performed using a FACS Aria II flow cytometer (BD Biosciences).

### Statistical analysis

The data were analyzed with Sigma Plot 10.0. Statistical differences between two groups were tested with a two-tailed Student’s *t* test. Data are represented as mean ± SEM. Survival data were analyzed with the Kaplan–Meier test. For all tests, *p < 0.05 was considered significant.

## Results

### CHIR and IDE1 inefficiently induced DE differentiation from hPSCs

Our previous studies and other reports have demonstrated that CHIR, a GSK-3β inhibitor that acts as a Wnt agonist, combined with Activin A could induce hPSCs efficient differentiation into DE [12, 17–19]. However, Activin A is an expensive growth factor, limiting its use in large-scale cell production. In order to replace Activin A, researchers have tried to screen available small molecules and found that IDE1 and IDE2 are the only two molecules that could partly replace Activin A for DE induction [7, 20–22]. Thus, we examined if IDE1 could replace Activin A, combined with CHIR, to direct DE differentiation efficiently, according to the Activin A plus CHIR-treated pattern (CA) (Fig. 1A).

Our results showed that this small-molecule cocktail (CI-I) failed to induce homogeneous DE-like cells morphology as CA (Additional file 2: Fig. S1). OCT4 and NANOG are essential transcription factors required to maintain the pluripotency and self-renewal of undifferentiated hPSCs. And SOX17 and Brachyury (BRA) are the markers of DE and MD, respectively. Thus, we analyzed the expression of these proteins to identify the cell fate determination and lineage differentiation. The immunostaining showed that the CI-I merely yielded 70% SOX17-positive cells, and plenty of cells remained OCT4 positive or appeared to express Brachyury (BRA), indicating mixed undifferentiated hPSCs and mesoderm (MD) cells (Fig. 1B). Therefore, we further optimized the small-molecule cocktail by supplying 1 μM CHIR on day 2 and day 3 (CI). The results showed that the optimized condition improved DE induction slightly, but still tricky to derive DE efficiently as CA formula (Fig. 1B).

Moreover, genes expressions of pluripotency, primitive streak (PS), primitive endoderm (PrE), and DE were dynamically traced daily during the differentiation process (Fig. 1C). The results showed a minor decrease in the pluripotency markers *OCT4* and *NANOG* expression levels. In contrast, DE markers *SOX17* and *FOXA2* increased lower in cells treated with CI-I or CI condition, compared with CA-treated group (Fig. 1D). Notably, the expression of PrE markers *SOX7* and *GATA6* has been highly upregulated in the process of CI-I or CI induction, indicating that PrE lineage was induced (Fig. 1D). Additionally, immunostaining showed the SOX7-positive cells induced by CI-I or CI condition further confirmed the genes expression profile (Fig. 1E). These results suggest that the combination of CHIR and IDE1 cannot induce DE efficiently, but derived an MD, PrE, DE lineages, and undifferentiated hPSCs mixed population.

During the differentiation process, the dynamic expression of crucial pathway-related genes was analyzed by RT-PCR. Results showed that the expression pattern of *Wnt3A* and *FGF8* has no significant difference among the groups. In contrast, *BMP2* expression was increased progressively after day 1, while *NODAL* remains a low-level expression in cells treated with CI-I and CI condition, compared with the CA treatment (Fig. 1F). These results suggested that lower endogenous Nodal signal and high level of BMP signal activities might be the reason why CI-I and CI treatment yielded a mixed population. Hence, increasing the endogenous TGF-β/Nodal signaling activity to improve current differentiation protocols potentially could be a reliable strategy for efficient DE differentiation.

### Manipulating small-molecule cocktails to improve DE differentiation

Inhibition of PI3K signaling pathway or cell-cycle regulators CDK4/6 have been reported to promote the phosphorylation of Smad2/3 and regulate the signal transduction of the Nodal pathway while inducing DE differentiation [23, 24]. Accordingly, we combined CHIR and IDE1 with the above candidate small molecules, including PI3K inhibitor LY294002 (Ly) and CDK4/6 inhibitor PD, to induce DE differentiation (Fig. 2A). During the differentiation process, morphological changes of the differentiated cells were monitored by microscopy, and phase-contrast images showed sequential morphologic changes of hPSCs differentiation into DE-like cells (Fig. S1). RT-PCR analyzed the dynamic expression of related genes, and results showed that pluripotency markers *OCT4* and *NANOG* downregulated significantly. In contrast, CILy and CIP induced higher DE markers SOX17 and FOXA2 expression levels *than* the CI treated alone. Notably, the highly upregulated expression pattern of PrE markers *SOX7* and *GATA6* was induced by CI condition not seen in CILy- and CIP-treated cultures (Fig. 2B). These genes expression patterns suggested that added Ly or PD could improve DE differentiation versus PrE differentiation.

Immunostaining with DE markers SOX17 and FOXA2 further confirmed that a higher proportion of DE population in CILy and CIP condition, especially CIP, efficiently generated a near homogenous SOX17 and FOXA2 co-expressed DE population, which comparable with CA treatment as a control (Fig. 2C). Further FACS analysis showed that only 68.32% of the cells co-expressing SOX17 and FOXA2 were induced by CI, while up to 85.12%, 98.15%, and 98.5% in the cells induced by CILy, CIP, and CA, respectively (Fig. 2D). These results indicate that the combination of small-molecule CILy and CIP can efficiently induce DE differentiation.

### Elevating endogenic TGF-β/Nodal signal to promote DE differentiation

**Fig. 3:**
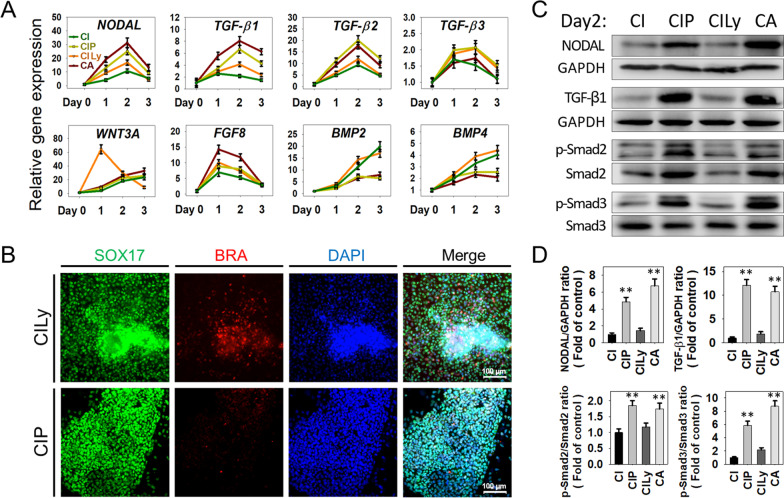
Elevating endogenic TGF-β/Nodal signal to promote DE differentiation. **A** Quantitative RT-PCR analyzed signaling pathway-related genes expression patterns in DE induction with different protocols. Data are presented as mean ± SEM, n = 3. **B** Immunostaining analyzed SOX17 and BRA expression in the CILy and CIP-derived differentiated cells. Scale bars 100 μm. **C** Representative immunoblots of NODAL, TGF-β1, and the phosphorylation of Smad2/3 at day 2 during DE induction with different protocols. **D** Quantification of the protein expression levels. Data were expressed as mean ± SD, n = 3 for each group, * *P* < 0.05, ** *P* < 0.01 vs control (CI) group

To test whether PD and Ly promoted the DE differentiation by regulating the transcriptional activity of TGF-β/Nodal-Smad2/3 signaling, we examined the expression profiles of genes related to TGF-β/Nodal, BMP, and Wnt signal pathways. Gene expression analysis showed a progressive induction of *NODAL, TGF-β1*, and *TGF-β2* by CIP, comparable with the CA control group but significantly higher than CI and CILy treatment. By contrast, the expressions of *BMP2* and *BMP4* were relatively lower in CIP and CA condition on the last two days of the differentiation process (Fig. 3A). These results indicate that the addition of PD could increase the endogenous TGF-β/Nodal signal while relatively downregulate BMP signal activity.

On the other hand, adding Ly increased significantly and accelerated the expression of *WNT3A*, with the expression peak appearing two days earlier and twice higher than other induction groups (Fig. 3A). As well known that Wnt is crucial to direct hPSCs differentiation and also induce MD formation. Thus, these results aligned with the above results that CILy rapidly reduced expression of pluripotent markers OCT4 and NANOG within 1-day induction while inducing the highest level of PS/MD marker gene *BRA* during the differentiation and accelerated *Mixl1* expression to transiently peak at day 1 (Fig. 2B). Further immunostaining showed that many BRA-positive cells were induced in CILy treatment at day 3, while few in CIP-treated condition (Fig. 3B). Moreover, these CILy-derived MDs could develop into contracting cardiomyocytes in the later phase of the hepatic differentiation process (Additional file 1: Movie.1). These results indicated that blocking the PI3K pathway with Ly amplifies the Wnt signaling, which accelerated hPSCs differentiation and MD induction.

Further analysis of protein expression by Western blot showed the effect of the different protocols on TGF-β/Nodal signaling molecules and downstream phosphorylation level of Smad2/3. Results showed that adding PD (CIP) increased the amounts of NODAL and TGF-β1 proteins significantly, while little change was observed in CILy-treated cells compared with CI treatment. In contrast, protein bands upon CA-treated cells revealed the highest expression level. Similarly, Smad2/3 and phosphorylated Smad2/3 (p-Smad2 and p-Smad2) showed a low protein expression in CI-treated cells while increased upon treatment with CIP and CA (Fig. 3C). Quantification of the protein bands verified the higher NODAL, TGF-β1 proteins, and stronger phosphorylation Smad2/3 of CIP-treated samples compared to the CI and CILy groups (Fig. 3D). Thus, we concluded that the addition of PD to the combination of CI could upregulate the endogenous TGF-β/Nodal signal and the downstream Smad2/3 transduction activities to promote the differentiation of DE.

### Small-molecule cocktail VDF directed hepatic specification

**Fig. 4:**
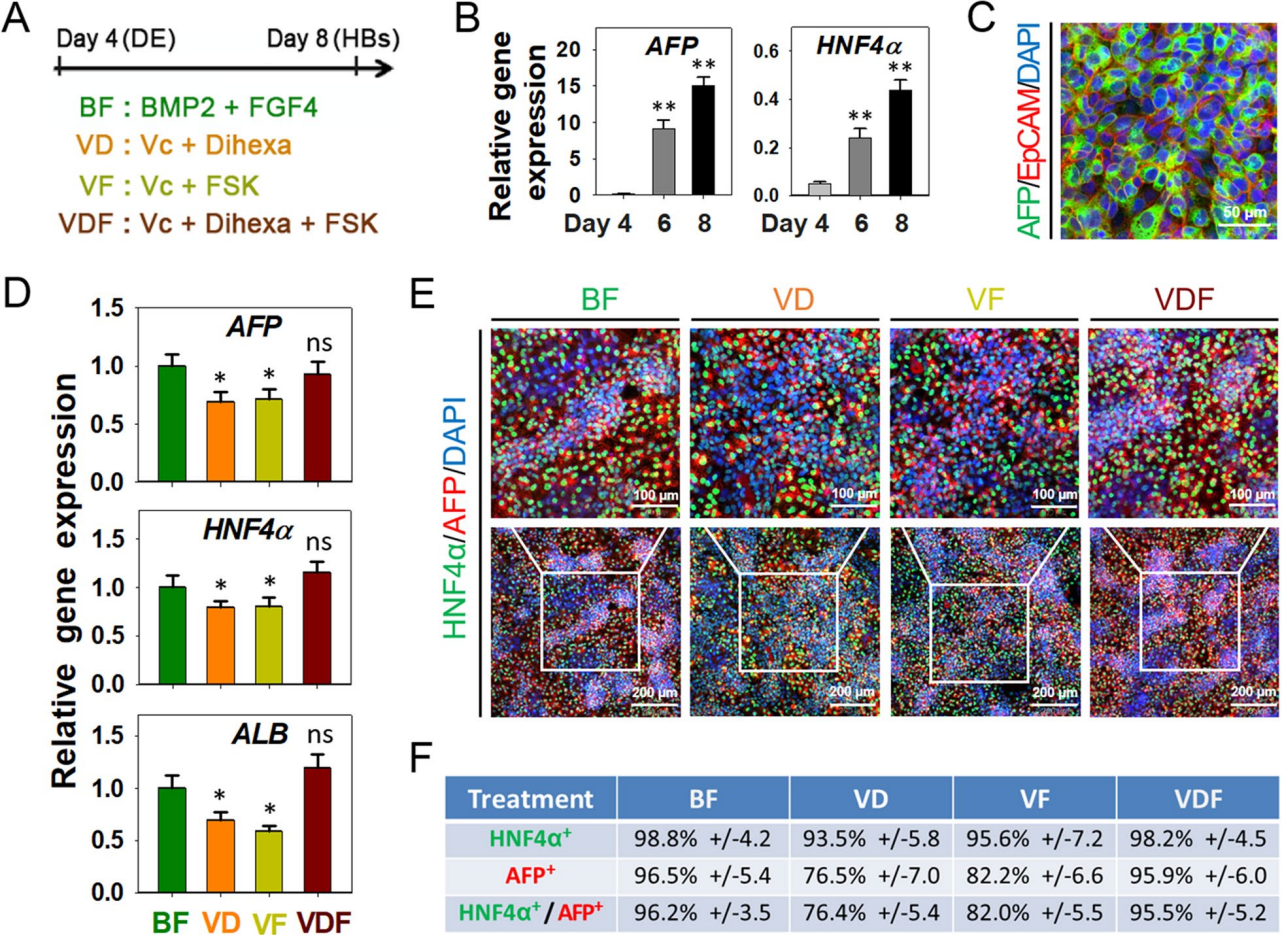
Small-molecule cocktail VDF directed hepatic specification. **A** Schematic of the strategy to identify chemical culture cocktails for HB specification. **B** RT-PCR analyzed *AFP* and *HNF4α* expression during hepatic specification. Data are presented as mean ± SEM, n = 3. **C** Immunostaining analyses of AFP and HNF4α expansion after 5 days of BF induction. Scale bars 100 μm. **D** RT-PCR results show hepatic genes expression in differentiated cells induced by different small molecule cocktails. Data are presented as mean ± SEM, n = 3. **E** Immunostaining analyses of AFP and HNF4α expression after different small molecule cocktails induced. **F** The efficiency of AFP and HNF4α expression after different chemical cocktails treatment, determined by counting positive cells. Efficiencies are presented as the percentage of positive cells plus or minus the SD of all fields counted

Further endodermal specifying a hepatic fate generally utilize growth factors including BMPs, FGFs, and HGF. To identify effective small molecules substitute growth factors for hepatic specification, we specifically explored the availability of Vc (previously used to replace BMPs in HBs expansion), the HGF agonist Dihexa, and Forskolin (FSK, an adenylate cyclase activator and thus activating cAMP/PKA signal transduction) in inducing a hepatic fate, since these small molecules have been shown to promote differentiation and proliferation of HBs in previous studies [6, 13, 25] (Fig. 4A).

We firstly induced CIP-derived DE cells into HBs, using the combination of BMP2 and FGF4 (BF). As expected, genes expression and immunostaining analyses showed the expression of HBs marker AFP and EpCAM after five days of induction (Fig. 4B, C), indicating that CIP-derived DE cells could be differentiated into HBs efficiently. Furthermore, we observed that exposure to either Dihexa or FSK in the presence of Vc resulted in induction of the hepatic marker genes *AFP*, *HNF4α*, and *Albumin* (*ALB*), with an expression rate slightly lower than the BF control group. In comparison, the triple combination of Vc, Dihexa, and FSK (VDF) results in the best overall induction of the three hepatic markers (Fig. 4D). Further immunostaining with AFP and HNF4α confirmed that cells treated with the combination of VDF highly co-expressed the hepatic markers (Fig. 4E, Additional file 2: Fig. S2), and the efficiency (95.5%) was comparable with the BF control group (96.2%) (Fig. 4F).

To further confirm the HBs differentiation methodology, we tested another hPSC line (iPSC line UC15) using the optimized small-molecule condition. The results showed that this iPSC line also could sequentially differentiate into DE cells and HBs, with high efficiency and expressed stages-specific markers (Fig. S3). These results indicated that the combination of small-molecule VDF could substitute growth factors to induce hepatic specification.

### Expansion and maintain of small-molecule-derived HBs

**Fig. 5:**
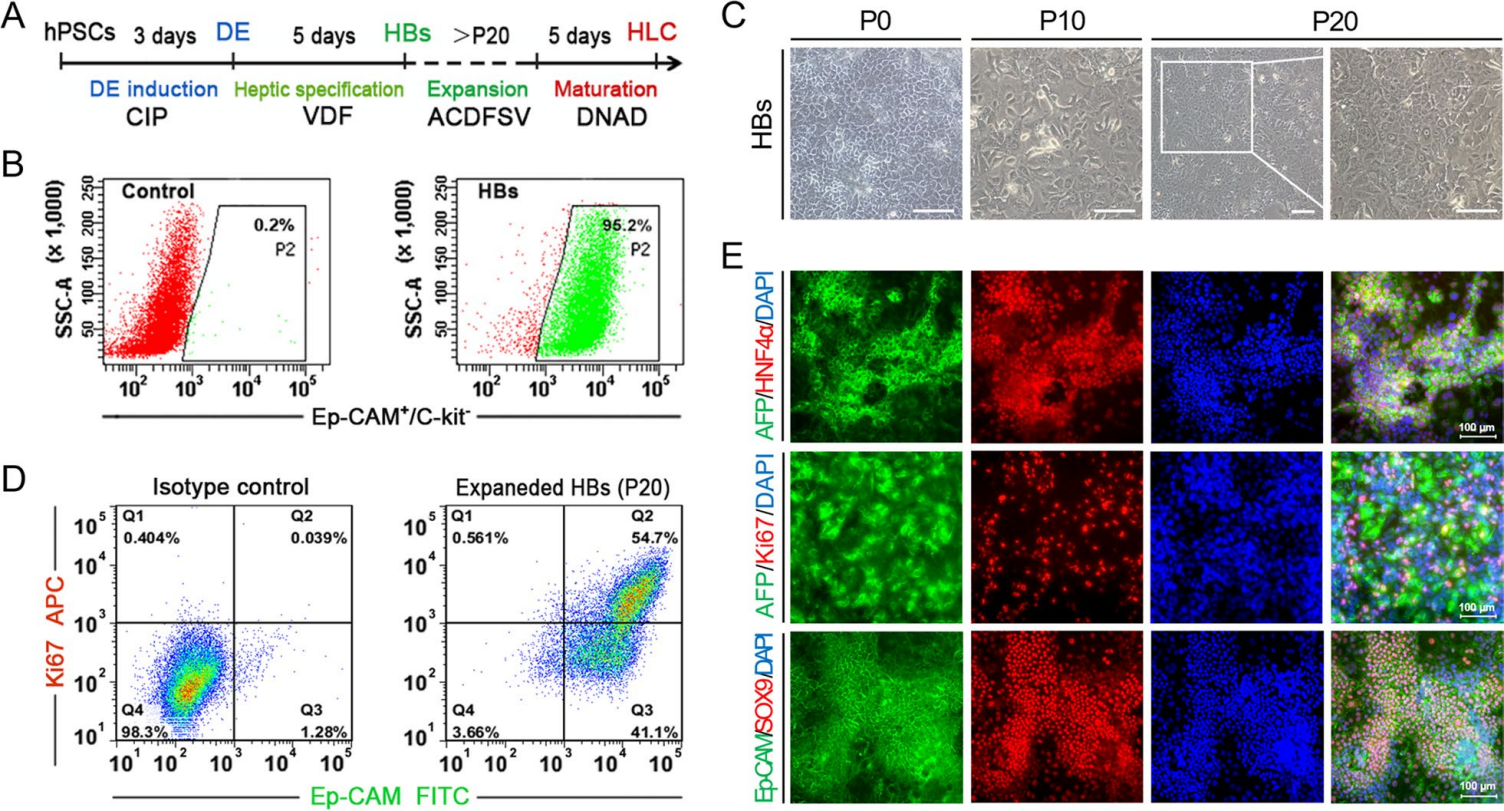
Expansion and maintain of small-molecule-derived HBs. **A** A schematic description of stepwise differentiates hPSCs into HBs and HLCs. **B** Flow cytometric sorting of E-pCAM^+^ HBs from day 8 differentiated cells. **C** Phase-contrast images of expanding HBs. Scale bar 100 μm. **D** Flow cytometric analyses of E-pCAM and Ki67 expression in expanded HBs (passage 20). **E** Immunostaining analyses of HBs marker proteins on expanded HBs. Scale bars 100 μm

Our previous study has established a robust and cost-effective culture condition for generating self-renewing HBs and functional mature HLCs, using a small-molecule cocktail in a defined culture system [13]. Based on this, we attempted to set up a complete small-molecule formula to efficiently generate expanded HBs and functional mature HLCs (Fig. 5A).

Firstly, we purified the HBs by cell sorting with EpCAM antibody, which is well known as a specific surface marker to isolate HBs (Fig. 5B). As expected, HBs populations were isolated from the differentiated cells (day 8) with a proportion of 95.2%, which is consistent with the ratio of small-molecule VDF-derived AFP and HNF4α double-positive HBs showing above. The purified HBs successfully expanded in the small-molecule culture condition (ACDFSV) with a reliable self-renewal capacity. Additionally, the proliferative HBs could be cultured stably for at least 20 passages, without significant morphologic change and an apparent decrease in proliferative capacity (Fig. 5C).FACS analysis showed that 54.7% EpCAM positive HBs (at passage 10) co-positive with proliferative marker Ki67 (Fig. 5D). Immunostaining further confirmed that expanding HBs almost co-expressed typical hepatic marker AFP and HNF4α. Moreover, considerable AFP-positive cells co-expressed with Ki67 (Fig. 5E), indicating the preferable proliferative capacity of HBs. Further results exhibited bipotency features in the proliferative HBs, with hepatic markers and early cholangiocyte markers SOX9 and Ep-CAM co-expression (Fig. 5E). During expansion, these cells could be frozen and thawed repeatedly.

### Differentiation of proliferative HBs into functional mature hepatocytes

**Fig. 6:**
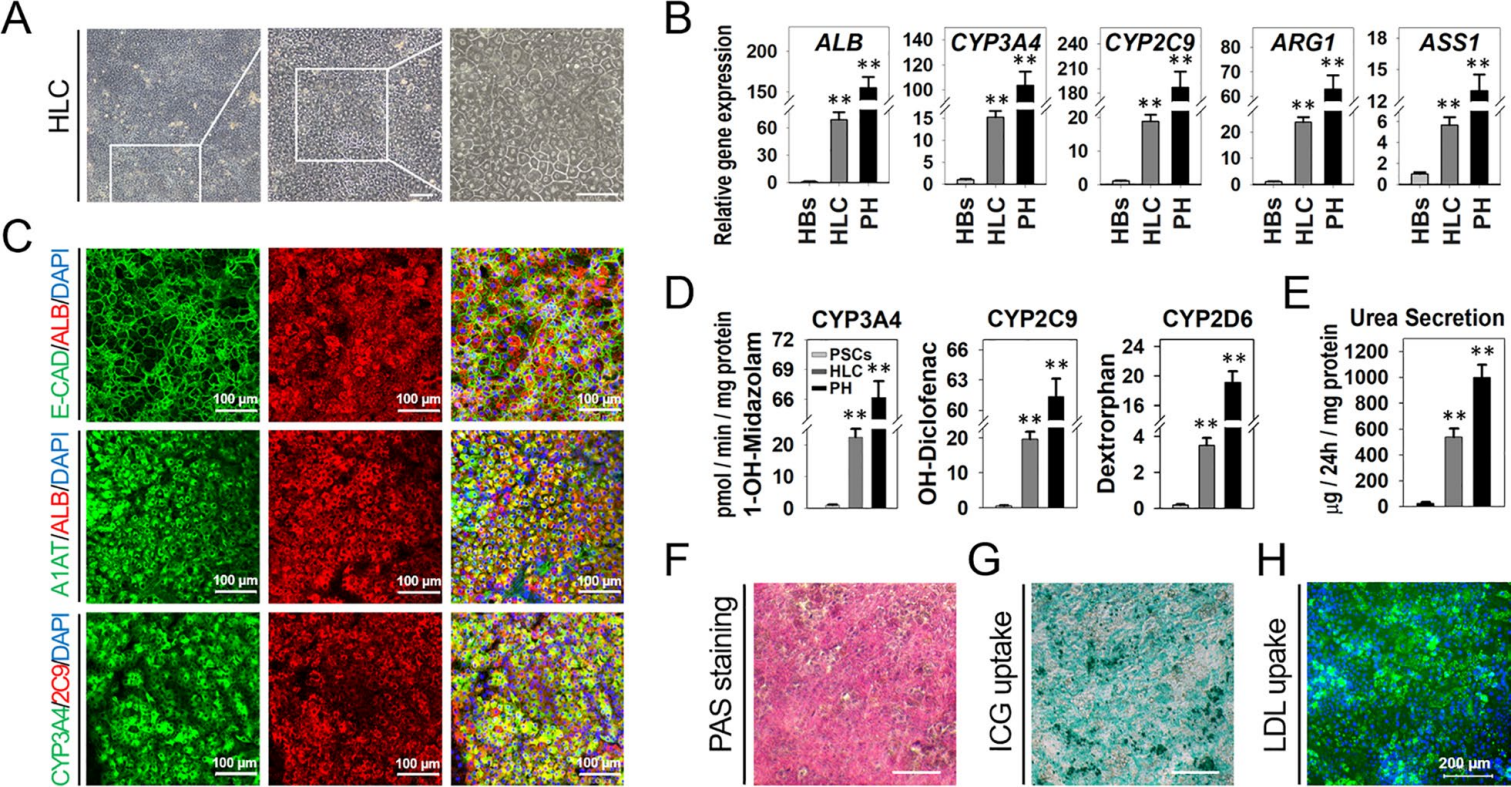
Differentiation of proliferative HBs into functional hepatocytes. **A** The morphology of small-molecule protocol-derived HLCs. HLCs exhibited polygonal shapes and distinct round nuclei; even some had two nuclei. Scale bar 100 μm. **B** Quantitative RT-PCR analyzed the genes expression levels of ALB, the CYP and urea cycle enzymes. Gene expression was normalized to HBs. Data are presented as mean ± SEM, n = 3. * *P* < 0.05, ** *P* < 0.01. **C** Immunostaining analyses of mature hepatocyte markers (ALB, A1AT, CYP3A4, and CYP2C9) on small-molecule protocol-derived HLCs. Scale bars 100 μm. **D** CYP450 activity assay of HLCs. Data are presented as mean ± SEM, n = 3. * *P* < 0.05, ** *P* < 0.01. **E** Urea secretion of HLCs was analyzed. Data are presented as mean ± SEM, n = 3. * *P* < 0.05, ** *P* < 0.01. **F** PAS staining on HLCs. Scale bar represents 200 μm. **G** ICG uptake analyses in HLCs. Scale bar represents 200 μm. **H** The HLCs were stained by Alexa-Flour 488-ac-LDL. Scale bar 200 μM

For hepatic maturation, the proliferative HBs then cultured in previously established small-molecule culture conditions, including A8301, Dex, Dihexa, and NH4Cl (DNAD) [13]. After five days of induction, differentiated cells displayed a homogenous and typical mature hepatocyte-like polygonal morphology (Fig. 6A). Genes expression of hepatic matured and relative functional biomarkers were analyzed to identify the derived HLCs. The results indicated that the *ALB* and cytochrome P450 (CYP) and urea cycle enzymes transcription were induced in the derived HLCs (Fig. 6B). Human PHs used as a control in the experiments. Immunostaining analyses further confirmed that the derived HLCs typically co-stained with mature hepatocyte-specific proteins ALB, A1AT, CYP3A4, and CYP2C9 (Fig. 6C).

Furthermore, we assessed the metabolism and detoxification functions of the small-molecule-derived HLCs by characterizing the activities of CYP enzymes. Three typical CYP isoforms (CYP3A4, CYP2C9, and CYP 2D6) were examined by measuring the increase in CYP isoform metabolites in response to exposure to their respective probe substrates. Results released considerable levels of CYP enzyme metabolic activities compared with that of human PHs (Fig. 6D). The HLCs also showed a high urea secretion pattern, corresponding to approximately 50% urea secreted by the human PHs (Fig. 6E). Moreover, periodic acid-Schiff staining (PAS), indocyanine green (ICG), and LDL uptake analyses showed that HLCs displayed cytoplasmic glycogen storage, ICG, and LDL uptake abilities (Fig. 6F-H). Taken together, these results indicated that the current small-molecule protocol could direct hPSCs to efficiently differentiate, expand, and maturate into metabolically functioning hepatocytes in vitro.

### Repopulation of mice injured liver by HBs transplantation

**Fig. 7:**
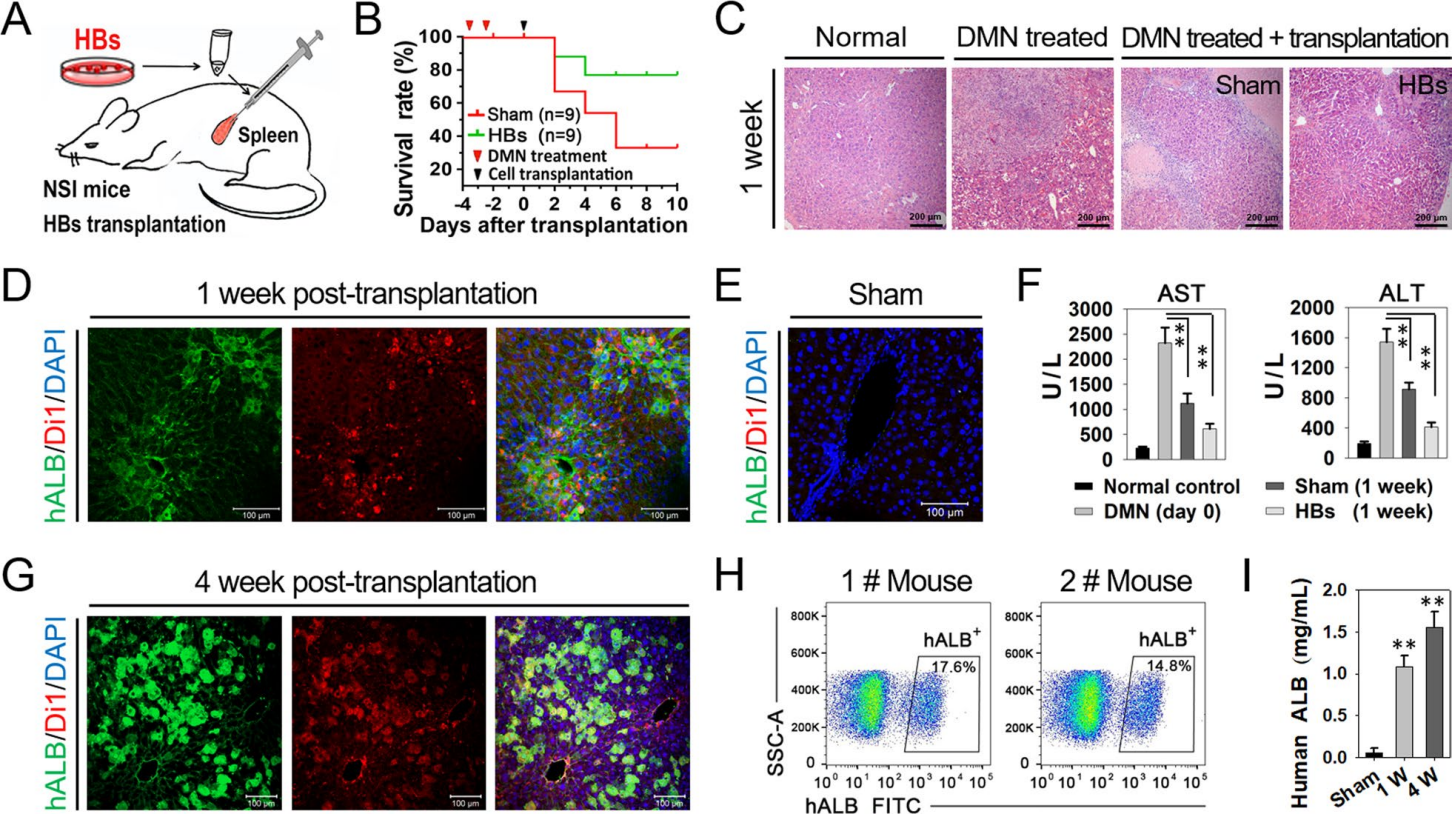
Repopulation of mice injured liver by HBs transplantation. **A** Schematic diagram depicting HBs transplantation experimental schedule in immune-deficiency mice **B** Survival curve of mice. **C** Hematoxylin and eosin staining in mice liver. Scale bar 200 μM. **D**, **E** Engraftment of transplanted human HBs in mice liver after 1 week transplantation, as indicated by immunostaining of human ALB (green) and Di1 (red). Sham mice's liver as the negative control. Scale bar 100 μM. **F** Detection of AST and ALT levels in mice serum at one week after HBs transplantation. Data were analyzed by 2-tailed t tests. **G** After 4 weeks of cell transplantation, the reproduction of mice liver is indicated by immunostaining of human ALB (green) and Di1 (red). Scale bar 100 μM. **H** Flow cytometric analyses human ALB-positive hepatocytes in mice liver transplanted with human HBs. **I** Human ALB secretion in the mice serum. Data are presented as mean ± SEM, n = 4

To determine whether the HBs could differentiate into functional hepatocytes and repopulate the injured liver in vivo, we transplanted HBs into DMN-induced acute liver failure of NSI mice (Fig. 7A). The Kaplan–Meier survival estimates were determined for ten days after cell transplantation. In the sham control group, two-thirds (6 of 9) of animals died within five days after the DMN injection while inducing acute liver failure. For the HBs transplanted groups, the survival rate was nearly 80% (7 of 9) throughout the examination period (Fig. 7B). Hematoxylin and eosin (H&E) staining revealed massive necrosis loci associated with inflammatory cell infiltration in the DMN-treated mouse liver, suggesting DMN-induced acute liver failure. In contrast, the necrosis loci dramatically decreased and morphologically nearly restored to normal control in the HBs transplanted mice liver (Fig. 7C).

Moreover, to trace the homing cells and analyze the repopulation efficiency, HBs were labeled with Di1 dye before transplantation. After one week of transplantation, immunostaining with a human ALB (hALB) antibody was conducted. Representative patterns of Di1-positive and hALB-positive cells in the HBs transplanted mice liver were shown (Fig. 7D). In contrast, no hALB-positive cells were detected in the sham control group (Fig. 7E). These results indicated that the small-molecule protocol-derived HBs could home and differentiate into mature hepatocytes after being transplanted in vivo. The levels of AST and ALT were significantly decreased in mice transplanted with HBs compared to the sham-operated mice (Fig. 7F). These results suggest that HBs transplantation benefits the parenchymal tissue and the damaged recipient liver's functional recovery.

Four weeks post-transplantation, Di1 and hALB co-positive cells were observed more widespread in HBs transplanted mice liver (Fig. 7G). We further analyzed the cell engraftment by the FACS examination of hALB-positive hepatocytes in the recipient's liver. Results showed that around 15% (14.8% and 17.6% in two mice liver, respectively) of the hepatocyte mass mightily derived from the transplanted human HBs, judging by the hALB labeled cells (Fig. 7H). Examination of human ALB in the mice’s serum showed increasing human ALB secretion from 1 week to four weeks after hepatic cell transplantation (Fig. 7I).

No teratomas or other tumor types were found in any transplanted recipients during a 4-week period. Therefore, we concluded that the current small-molecule protocol-derived HBs could engraft and differentiate into functional hepatocytes, which further attains injured parenchyma tissue repopulation in the recipient’s liver after transplantation.

## Discussion

This study established an efficient protocol for the specification, expansion, and maturation of hepatic cells from hPSCs, using small molecules in a chemical defined culture system. In developing the protocol, we first demonstrated that the combination of CIP could efficiently induce DE differentiation via cell cycle inhibition by PD to increase endogenous TGF-β/Nodal signaling. Furthermore, we identified that combining Vc, Dihexa, and FSK (VDF) could substitute growth factor BMPs, FGFs, and HGF to induce hepatic specification. In addition, the small-molecule-derived HBs subsequently expanded and matured into functional HLCs by the strategy established in our previous study. The generated HLCs displayed adult hepatocyte's typical functional characteristics in vitro and the ability to repopulate injured liver in vivo. Thus, the current full small-molecule-based hepatic generation protocol presents an efficient and cost-effective strategy for the large-scale production of self-renewal HBs and functional mature hepatocytes for cell-based therapy and drug discovery use.

DE induction is the first stage also a critical stage of hPSCs differentiation into hepatic cells. The efficacy of DE induction and their heterogeneity influenced the following hepatic differentiation trajectory and outcome significantly [23, 26]. Although applying a high concentration of Activin A combined with Wnt3a or Wnt agonist CHIR could efficiently differentiate hPSCs into DE [17, 19, 27], while Activin A is an expensive growth factor, limiting its use in large-scale cell production. Small-molecule methods yielded low efficiency and reproducibility differentiation [8, 15, 22, 28], which may be due to no available small molecule that can efficiently substitute Activin A to induce DE differentiation. Melton et al. have reported that IDE1/2 can phosphorylate Smad2/3 and activate Activin A /Nodal signaling to induce hPSCs into the endodermal lineage [20]. However, IDE1 treated alone failed to efficiently generate DE in our preliminary studies (data not shown), and similar results (40–80% SOX17-positive cells) were also reported in other studies [7, 8, 22, 28].

Further combining CHIR with IDE1 (CI-I and CI methods) improved the differentiation significantly but still yielded a mixture population, including DE, undesired MD and PrE lineage, and also with undifferentiated hPSCs. Both CI-I and CI treatment induced PrE marker SOX7 expression while barely expressed in CA-induced DE differentiation. Previously reported that low Nodal and high BMP signals would lead to the upregulation of SOX7 and PrE differentiation [29–31]. Thus, inefficient differentiation outcome may be due to insufficient endogenous Nodal signal while a high level of BMP signal activities stimulated by CI-I or CI, compared with CA treatment. That highlights the critical roles of endogenous signals in DE differentiation. Therefore, we attempted to increase endogenous TGF-b/Nodal signaling activity by manipulating the small-molecule cocktails to improve DE differentiation.

PI3K signaling pathway acted as a threshold at the initial stage of hPSCs differentiation [23, 32, 33]. The activation and transduction of Nodal and Wnt signaling pathways require decreased PI3K activity to trigger hPSCs differentiation [33]. Similarly, cell-cycle regulators CDK4/6 also have been reported to regulate the phosphorylation of Smad2/3 and control the signal transduction of the Activin A/Nodal pathway [24]. Moreover, inhibiting PI3K or CDK4/6 with small molecules promoted Smad2/3 to bind and activate DE genes while inducing DE differentiation. Hence, we combined CI with candidate small molecules, including PI3K inhibitor LY and CDK4/6 inhibitor PD, to induce DE differentiation.

Our finding demonstrated that adding PD to CI treatment (CIP) improved DE gene expression while dismissing *SOX7* upregulation, resulting in efficient DE formation. Further results released that adding PD increased endogenous TGF-β superfamily genes and proteins (Nodal and TGF-β1) expression significantly while downregulating the transcription of *BMP2* and *BMP4.* Furthermore, the addition of PD also increased the phosphorylation of Smad2/3 (p-Smad2 and p-Smad2), which is comparable with CA treatment. These results aligned with previous reports that PD could induce endogenous Nodal signaling in hPSCs differentiation [24]. Therefore, we concluded that adding PD to CI can upregulate endogenous TGF-β/Nodal signaling and downstream Smad2/3 transduction activities while relatively downregulating BMP signal activity, which leads to the promotion of DE fate versus MD/PrE differentiation.

Moreover, we found that the addition of Ly to CI treatment (CILy) led to exceeding amplification of Wnt signal, consistent with previously reported that PI3K negatively regulates the activity of the Wnt signal pathway [33, 34]. However, high Wnt signal activity tends to specify a ME formation [35–37], thereby affecting the differentiation of DE. This might account for why CILy treatment led to the highest MD marker gene *BRA* level and induced BRA-positive MD lineages during the differentiation process while yielding lower efficiency DE formation.

Small molecules have been identified to promote DE induction, while few chemicals have been reported to support hepatic specification effectively. Further specifies DE to a hepatic fate in previous small-molecule-based protocols mainly relied on sequential treatment with sodium butyrate and DMSO, which played a part in epigenetic modifications and were associated with ambiguous hepatic induction [15, 17, 38]. However, variable studies reported whether these nonspecific agents are sufficient for hepatic differentiation. Rambhatla et al. indicated that DMSO was insufficient to differentiate DE cells into hepatic lineage [39]. In addition, both DMSO and sodium butyrate inhibit cell growth, which might compromise the yield of hESCs-derived hepatocytes for applications. Yet, no report indicated effective small molecules substitute for BMPs and FGFs to direct hepatic specification efficiently. Thus, identifying novel small molecules with hepatic-specific induction would contribute to the strategy of functional hepatic cells production.

Our recent study has identified that Vc, FSK, and Dihexa could replace BMP4, EGF, and HGF, respectively, supporting HBs expansion and maintenance [13]. Also, we demonstrated that Vc could phosphorylate BMP downstream Smad1/5/8, consistent with the previous report that Vc serves a similar effect in cardiomyogenesis [40]. Besides, FSK has been demonstrated essential for downregulating EMT marker gene expression and sustaining PHs gene expression [25]. Moreover, Dihexa was identified in a screen that assessed the capacity to potentiate the biological activity of HGF and can act as a potent HGF agonist [6, 41], which could mimic the function of HGF in HBs differentiation and expansion. These results suggested the potential of Vc, Dihexa, and FSK to substitute growth factors for hepatic specification. In the present study, we showed that treatment with Vc and FSK combined with Dihexa (VDF) resulted in high expression of hepatic genes and proteins (AFP and HNF4α), comparable with growth factors-treated group as control. These results indicated that the combination of small-molecule VDF could substitute BMPs, FGFs, and HGF to induce hepatic specification efficiently.

HBs have capable of self-renewal and potency to differentiate into hepatocytes. Hence expanding HBs, especially in small-molecule culture conditions to scalable expand HBs derived from hPSCs would be an ideal strategy for efficient, large-scale, and cost-effective generating hepatic cells for the potential clinical application [12, 42]. According to this view, our previous study has established a robust and cost-effective small-molecule culture condition for the generation of self-renewing HBs and functional mature HLCs [13]. Thus, we then adopted the previously established culture condition to expand the small-molecule-derived HBs. Our finding released that the purified HBs could successfully expand in the proliferative culture condition (ACDFSV), with a reliable self-renewal capacity and hepatic properties maintained. Additionally, the proliferative HBs could undergo long-term culture for at least 20 passages without significant morphologic change and an obvious decrease in their proliferative capacity.

Moreover, the proliferative HBs could be further efficiently differentiated into functional mature HLCs after being induced with mature conditions (DNAD). The small-molecule-derived HLCs expressed various representative markers and exhibited typical function activities. Remarkably, the metabolic activity of CYP enzymes was comparable to PHs. In addition, after being transplanted into NSI mice with acute liver failure, these hepatic cells could engraft and differentiate into hepatocytes, resulting in considerable liver repopulation and functional recovery. These results indicated that our small-molecule-based hepatic induction method induces acquired hepatocytes to function in vitro and in vivo.

## Conclusions

In summary, we have developed a robust and cost-effective strategy for inducing hPSCs to proliferative HBs and functional hepatocytes using the small-molecule protocol, which could serve as a powerful foundation for future studies to define culture conditions using GMP-compatible products. Furthermore, this work provides a novel, cost-effective way for generating large-scale and functional human hepatic cells, which would result in an invaluable impact in the cell-based therapy industry.

## Supplementary Information

**Additional file 1:** CILy-derived MDs developed into contracting cardiomyocytes during hepatic differentiation.

**Additional file 2: Figure S1**. Small-molecule cocktails induced DE differentiation from hPSCs. Sequential morphologic changes in the differentiation of hPSCs into DE. Scale bars 100 μm. **Figure S2**. Small-molecule cocktails directed hepatic specification. Immunostaining analyses of AFP and HNF4α expression after different small molecule cocktails induced. **Figure S3**. Generation of HBs from human iPSC line. Immunostaining analyses results showed that human iPSC line (UC15) could sequentially differentiate into DE cells and HBs with high efficiency similarity as previous hESC line H1, and expressed stage specific markers.

## Data Availability

The datasets supporting the conclusions of this article are included within the article.

## Abbreviations

hPSCs: Human pluripotent stem cells
HBs: Hepatoblasts
HLCs: Hepatocyte-like cells
PHs: Primary hepatocytes
DE: Definitive endoderm
PrE: Primitive endoderm
NSI mice: NOD-SCID-IL2RG^−/−^ mice
CHIR: CHIR99021
CYP: Cytochrome P450
ICG: Indocyanine green
PAS: Periodic acid-schiff staining
