# Supplementary material for: Efficiently generate functional hepatic cells from human pluripotent stem cells by complete small-molecule strategy
Source: Stem Cell Res Ther. 2022 Apr 11;13:159. doi: 10.1186/s13287-022-02831-1 (PMC8996222; doi:10.1186/s13287-022-02831-1)
Supplement: Supplementary file 2 — Additional file 2: Figure S1. Small-molecule cocktails induced DE differentiation from hPSCs. Sequential morphologic changes in the differentiation of hPSCs into DE. Scale bars 100 μm. Figure S2. Small-molecule cocktails directed hepatic specification. Immunostaining analyses of AFP and HNF4α expression after different small molecule cocktails induced. Figure S3. Generation of HBs from human iPSC line. Immunostaining analyses results showed that human iPSC line (UC15) could sequentially differentiate into DE cells and HBs with high efficiency similarity as previous hESC line H1, and expressed stage specific markers. [file 13287_2022_2831_MOESM2_ESM.docx]

**Supporting information**

**Efficiently generate functional hepatic cells from human pluripotent stem cells by complete small-molecule strategy**

Tingcai Pan ^1, 2, †^, Ning Wang ^2 †^, Jiaye Zhang ^2 †^, Fan Yang ^3^, Yan Chen ^2^, Yuanqi Zhuang ^2^, Yingying Xu ^2^, Ji Fang ^2^, Kai You ^2^, Xianhua Lin ^2^, Yang Li ^1^, Shao Li ^1^, Kangyan Liang ^1^, Yin-xiong Li ^2,^ * and Yi Gao ^1, 4,^ *

1 General Surgery Center, Department of Hepatobiliary Surgery II,Guangdong Provincial Research Center for Artificial Organ and Tissue Engineering, Guangzhou Clinical Research and Transformation Center for Artificial Liver, Institute of Regenerative Medicine, Zhujiang Hospital, Southern Medical University, Guangzhou, Guangdong Province, China

2 Key Laboratory of Regenerative Biology, South China Institute for Stem Cell Biology and Regenerative Medicine, Guangdong Provincial Key Laboratory of Biocomputing, Guangzhou Institutes of Biomedicine and Health, Chinese Academy of Sciences, Guangzhou, 510530, China

3 Guangdong Key Laboratory of Non-Human Primate Models, Guangdong-Hongkong-Macau Institute of CNS Regeneration, Jinan University, Guangzhou, Guangdong Province, China

4 State Key Laboratory of Organ Failure Research, Southern Medical University, Guangzhou, China

† These authors contributed equally to this work.

* Correspondence: li_yinxiong_iph@gibh.ac.cn and gaoyi@smu.edu.cn.

**Supplementary figures**

**
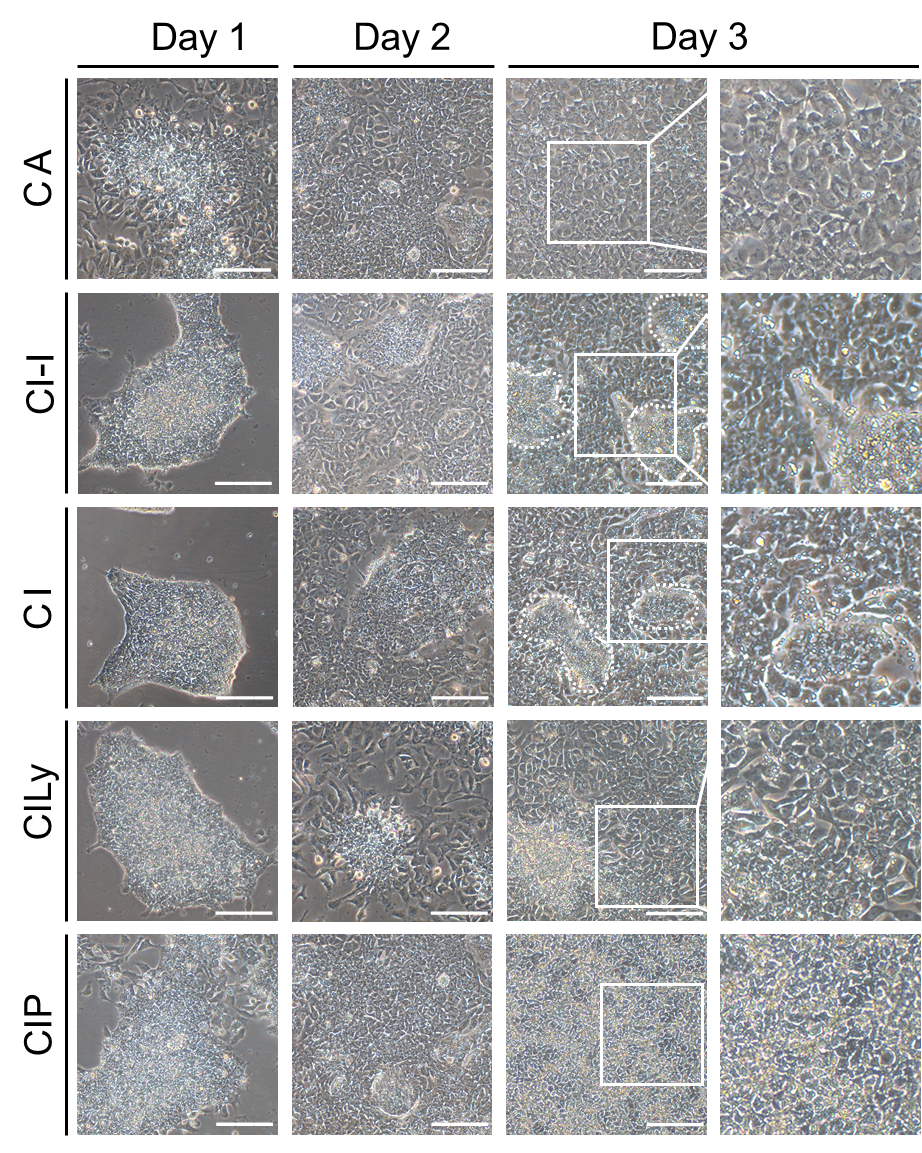
**

**Fig. S1. Small-molecule cocktails induced DE differentiation from hPSCs**

Sequential morphologic changes in the differentiation of hPSCs into DE. Scale bars 100 μm.


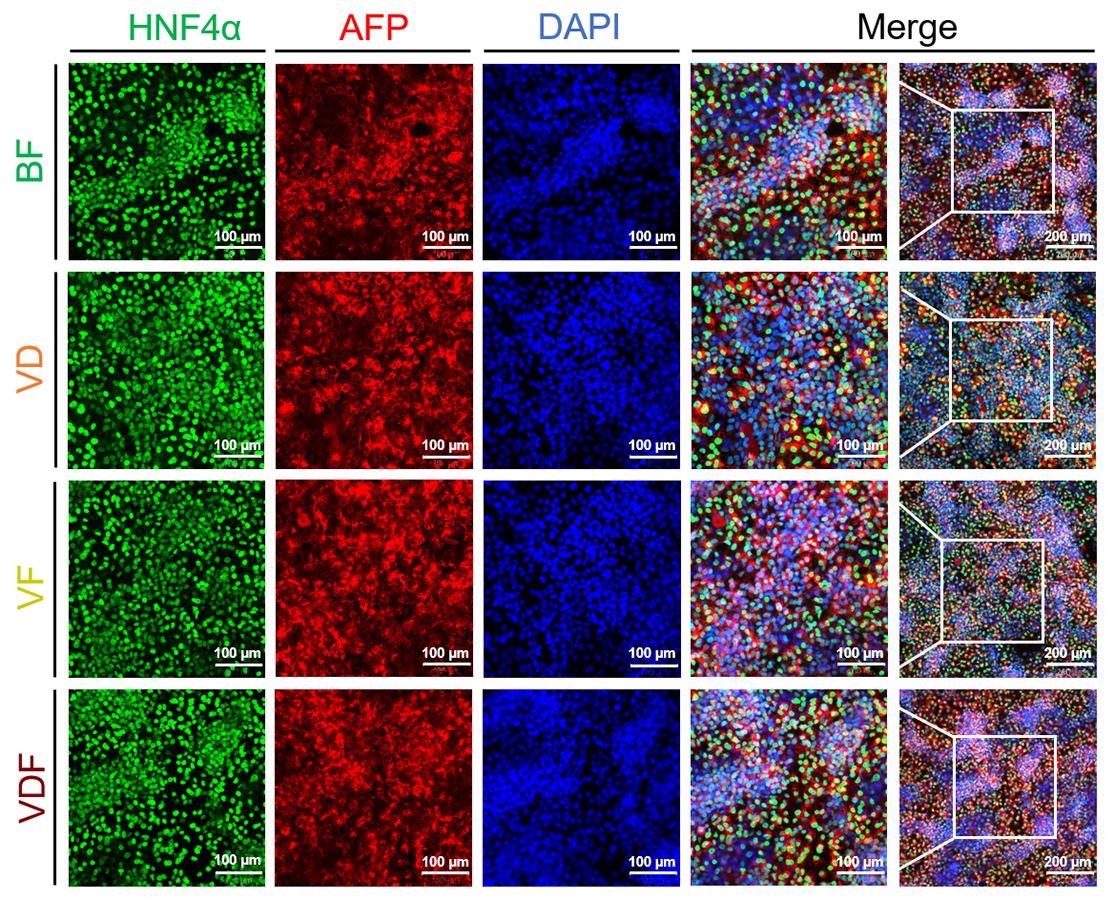


**Fig. S2. Small-molecule cocktails directed hepatic specification**

Immunostaining analyses of AFP and HNF4α expression after different small molecule cocktails induced.


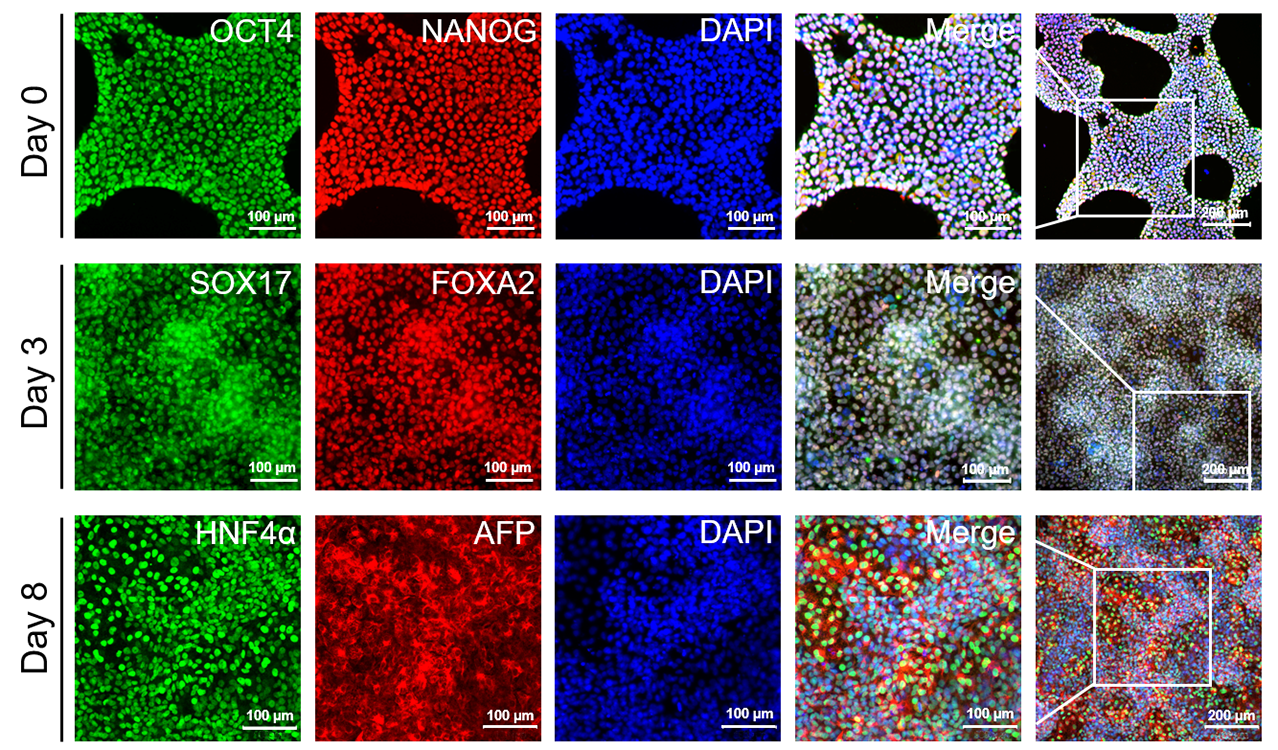


**Fig. S3. Generation of HBs from human iPSC line**

Immunostaining analyses results showed that human iPSC line (UC15) could sequentially differentiate into DE cells and HBs with high efficiency similarity as previous hESC line H1, and expressed stage specific markers.

**Supplementary Tables**

**Table 1. List of antibodies used in this study**

| **Gene** | **Primer sequence (forward / reverse; 5' to 3')** |
| --- | --- |
| *OCT4* | CCTCACTTCACTGCACTGTA / CAGGTTTTCTTTCCCTAGCT |
| *NANOG* | TGAACCTCAGCTACAAACAG / TGGTGGTAGGAAGAGTAAAG |
| *BRA* | TATGAGCCTCGAATCCACATAGT / CCTCGTTCTGATAAGCAGTCAC |
| *Mixl1* | GGCGTCAGAGTGGGAAATCC / GGCAGGCAGTTCACATCTACC |
| *GATA6* | GTGCCCAGACCACTTGCTAT / CCCTGAGGCTGTAGGTTGTG |
| *SOX7* | TCGACGCCCTGGATCAACT / CTGGGAGACCGGAACATGC |
| *SOX17* | GTGGACCGCACGGAATTTG / GGAGATTCACACCGGAGTCA |
| *FOXA2* | GGAGCAGCTACTATGCAGAGC / CGTGTTCATGCCGTTCATCC |
| *WNT3A* | GCCCCACTCGGATACTTCTTACT / GAGGAATACTGTGGCCCAACA |
| *FGF8* | GACCCCTTCGCAAAGCTCAT / CCGTTGCTCTTGGCGATCA |
| *BMP2* | ACTACCAGAAACGAGTGGGAA / GCATCTGTTCTCGGAAAACCT |
| *BMP4* | TAGCAAGAGTGCCGTCATTCC / GCGCTCAGGATACTCAAGACC |
| *NODAL* | CTGCTTAGAGCGGTTTCAGATG / CGAGAGGTTGGAGTAGAGCATAA |
| *TGF-β1* | CTAATGGTGGAAACCCACAACG / TATCGCCAGGAATTGTTGCTG |
| *TGF-β2* | CCCCGGAGGTGATTTCCATC / GGGCGGCATGTCTATTTTGTAAA |
| *TGF-β3* | ACTTGCACCACCTTGGACTTC / GGTCATCACCGTTGGCTCA |
| *AFP* | AGAACCTGTCACAAGCTGTG / GACAGCAAGCTGAGGATGTC |
| *HNF4α* | TGTACTCCTGCAGATTTAGCC / CTGTCCTCATAGCTTGACCT |
| *ALB* | TGCAACTCTTCGTGAAACCTATG / ACATCAACCTCTGGTCTCACC |
| *CYP3A4* | TTCAGCAAGAAGAACAAGGACAA / GGTTGAAGAAGTCCTCCTAAGC |
| *CYP2C9* | CTACAGATAGGTATTAAGGACA / GCTTCATATCCATGCAGCACCAC |
| *ARG1* | TGGACAGACTAGGAATTGGCA / CCAGTCCGTCAACATCAAAACT |
| *ASS1* | AGGAAAGGGGAACGATCAGGT / GTGTTGCTTTGCGTACTCCA |
| *GAPDH* | CAAAGTTGTCATGGATGACC / CCATGGAGAAGGCTGGGG |

**Table 2. List of primers for real-time PCR**

| **Primary anbibody** | **Species** | **Company** | **Cat Number** | **Dilution** |
| --- | --- | --- | --- | --- |
| OCT4 | Mouse | Genetex | GTX627423 | 1:200 |
| Nanog | Rabbit | Genetex | GTX100863 | 1:200 |
| FOXA2 | Goat | R&D systems | AF2400 | 1:200 |
| SOX17 | Mouse | Genetex | GTX83580 | 1:100 |
|  | Goat | R&D systems | IC1924A | 1:50 |
| BRA | Goat | R&D systems | AF2085 | 1:200 |
| SOX7 | Goat | R&D systems | AF2766 | 1:200 |
| AFP | Mouse | Genetex | GTX84948 | 1:200 |
| HNF4α | Rabbit | Genetex | GTX62347 | 1:200 |
| Ki67 | Rat | Thermo Fisher | 11-5698-82 | 1:100 |
|  | Rabbit | Genetex | GTX16667 | 1:200 |
| Ep-CAM | Mouse | Invitrogen | 53-8326-42 | 1:100 |
|  | Mouse | Abcam | ab46714 | 1:100 |
| ALB | Goat | R&D systems | MAB1455 | 1:200 |
|  | Rabbit | Dako | F0117 | 1:50 |
| SOX9 | Mouse | Abcam | ab76997 | 1:200 |
| E-CAD | Goat | R&D systems | AF648 | 1:200 |
| AAT | Rabbit | Abcam | AB166610 | 1:200 |
| CYP3A4 | Mouse | Genetex | GTX60577 | 1:200 |
| CYP2C9 | Rabbit | Genetex | GTX55583 | 1:200 |
|  | | | | |
| **Second antibody** | **Species** | **Company** | **Cat. Number** | **Dilution** |
| Anti-Goat IgG Antibody, Alexa Fluor 488 | Donkey | Invitrogen | A-11055 | 1:500 |
| Anti-Goat IgG Antibody, Alexa Fluor 568 | Donkey | Invitrogen | A-11057 | 1:500 |
| Anti-Mouse IgG Antibody, Alexa Fluor 488 | Donkey | Invitrogen | A32766 | 1:500 |
| Anti-Mouse IgG Antibody, Alexa Fluor 568 | Donkey | Invitrogen | A10037 | 1:500 |
| Anti-Rabbit IgG antibody Alexa Fluor 488 | Donkey | Invitrogen | A-21206 | 1:500 |
| Anti-Rabbit IgG antibody Alexa Fluor 568 | Donkey | Invitrogen | A10042 | 1:500 |
